# To the Editors of the Medical and Physical Journal

**Published:** 1805-01-01

**Authors:** R. Williams

**Affiliations:** Surgeon, Royal Navy. H. M. S. Northumberland, off Ferrol


### *To the Editors of the Medical and Physical Journal.*

GENTLEMEN,

IT being known at Madrid that Dr. Joseph Alcaraz, of Alicante, made use of oil for the cure of the yellow fever, a letter was written to him by order of the supreme Tribunal of Health there, desiring information; and his answer was published in the Madrid Gazette of Tuesday the 30th of October last, of which the following is a copy, translated by Mr. Hunter, British Consul General there.

The instant that patients are found to be attacked with the disorder, I order them to be rubbed with common oil, for about five minutes, over the whole body, except the breast and face; in this operation about half a pound of oil is expended; then I make them drink two cups of infusion of elder-flower made like tea, and to be covered up warm, shutting the windows, and fumigating the room with thyme. Every three hours I order them a cup of broth, and in the intermediate space the infusion of elder-flowers. This method is continued until a copious sweat comes on, which is generally followed by a bilious diarrhœa; and when the diarrhœa does not come on spontaneously, I endeavour to promote it by clysters of sea water. By these two evacuations I have cured the disorder in the two first days, without its passing to the second period, in all the forty-five patients whose names are annexed, and without any other remedy thereafter but tincture of bark to those who remained weak. I have observed further, that those frictions are not so sure a remedy to those patients who have entered into the second period of the disorder, which is commonly the third or fourth day, as the nervous system is then affected; but, at the same time, I have not failed of curing many of those who were in a very advanced period of the disease, and in whom the terrible symptoms of black vomit, hæmorrhage, and convulsions had already appeared. The forty-five persons whose names are mentioned, are those cured in the first period, without any other remedy but the friction of oil, without including many others cured in other periods, whose names have escaped me from my continual occupations.

Here follows the names, and after that a certificate by a Deputy of the Health Office at Alicante.

Copy, signed J. H. Consul General, Madrid.

As the names would only be superfluous, I have here omitted them; and I will beg to acquaint you, that this oleous friction of Dr. Joseph Alcaraz, is not by any means a new mode of treating the yellow fever; I was an eye witness of its being put in practice eight years ago by the late Dr. Douglas White, a surgeon in the British Navy, and, at that time, of his Majesty's ship Scipio, on the West-India station. The said gentleman was passenger in the Quebec frigate, of which I was surgeon, from Jamaica to Portsmouth, in the year 1797, and I had frequent opportunities of knowing his sentiments on the subject, which he placed implicit confidence in. From an experience of five years and upwards in the West-Indies (and at the time that fatal disease raged with its greatest violence) I will presume to say, that the oleous friction could never be depended on, and that patients trusted solely to its specific qualities would inevitably sink. It will most certainly prevent the admission of air to the surface, and by that means assist in promoting a diaphoresis, which in the first stage of that epidemic malady is generally a very favourable symptom.

I am, &c.

R. WILLIAMS, Surgeon, Royal Navy.

*H. M. S. Northumberland, off Ferrol, Nov.* 10, 1804.

---

